# Selection for specific behavioural traits does not influence preference of chasing motion and visual strategy in dogs

**DOI:** 10.1038/s41598-022-06382-6

**Published:** 2022-02-11

**Authors:** Judit Abdai, Ádám Miklósi

**Affiliations:** 1 MTA-ELTE Comparative Ethology Research Group, Budapest, Hungary; 2 Department of Ethology, Eötvös Loránd University, Budapest, Hungary

**Keywords:** Animal behaviour, Perception

## Abstract

Perception of inanimate objects as animate based on motion cues alone seems to be present in phylogenetically distant species, from birth (humans and chicks). However, we do not know whether the species’ social and ecological environment has an influence on this phenomenon. Dogs serve as a unique species to investigate whether selection for specific behavioural traits influences animacy perception. We tested purebred companion dogs, and assigned them into two groups based on the type of work they were originally selected for: (1) Chasers, tracking and chasing prey; (2) Retrievers, mark and remember downed game. We displayed isosceles triangles presenting a chasing pattern vs moving independently, in parallel on a screen. We hypothesised that Chasers prefer to look at chasing and Retrievers eventually focus their visual attention on the independent motion. Overall, we did not find a significant difference between groups regarding the looking duration of dogs or the frequency of their gaze alternation between the chasing and independent motions. Thus it seems that selection for specific traits does not influence the perception of animate entities within the species.

## Introduction

Certain motion characteristics elicit the perception of inanimate objects as animate^1,2^. It has been found that perception of animacy is already present at birth in human infants^3,4^ and chicks (*Gallus gallus*)^5,6^, indicated by their preference to self-propelled motion, including starting from rest and changes in speed. Thus, there seems to be an innate sensitivity to certain motion cues indicative of animacy that is present in phylogenetically distant species. Although, animacy perception may be widespread among animals, we have sparse knowledge about the motion cues triggering it, and whether and how the social and ecological environment of the species influences the manifestation of this phenomenon.

Chasing motion has been applied to investigate animacy perception in humans because it has several characteristics that may elicit the perception of moving objects as animate and the characteristics of the motion can be manipulated systematically^2,7^. Although it should be noted that this motion pattern may go beyond animacy perception. For example, due to goal-directedness in the motion of the chaser, the moving objects may be identified as agents^7–9^. Human infants from 3 months of age already discriminate between a chasing and independent motion, but only at 5 months of age they display similar behaviour as adults, looking longer at the independent one^10^. Researchers also showed how specific features influence perception of the chasing motion in human infants^11,12^ and in adults^2,7,12–14^.

Only a few studies have investigated animacy perception (or perception of chasing) in non-human species (e.g.^6,8,15^). Thus, it is difficult to reveal its evolutionary background, for example, whether selection for different social and ecological environment influences this type of perception. Comparative investigations could provide us with more insight, but it would be difficult to control for all potentially confounding factors (e.g. feeding strategy, natural habitat, environment during development, solitary vs group living lifestyle). Dogs have an advantage to study the perception of chasing motion due to the large within-species variability which allows to test whether selection for specific behavioural traits influence perception. Although the visual system of dogs in many ways preserved the characteristics of the vision of their ancestor^16^, there are also marked differences among breeds which are probably coupled with the traits (tasks) they were selected for^17,18^. This resulted in, for example, differences in skull length influencing the distribution of ganglion cells in the retina and the visual field including e.g. depth perception^17,19^. However, we have no information on whether such selection had an effect on more specific visual perception skills, including social perception, and whether it influences visual strategies applied by the different breeds.

In previous studies, we found that dogs, similarly to humans, perceive inanimate objects as animate based simply on their motion^8,9,20^, but species differences were also detected^9^. In two studies, we displayed to dogs simultaneously a chasing and independent motion side-by-side on a screen using geometric figures. Eventually both dogs and humans turned their visual attention to the independently moving figures. Preference for the independent pattern might emerge over time to explore the unfamiliar pattern after the rapid encoding of the features of the chasing pattern^8,9^ (see also^10^). However, the two species displayed different initial behaviour when we used different figures. In case of dots, both dogs and humans looked equally long at both patterns in the first half of the display^8^. We expected that changing the figures to isosceles triangles (representing a bilateral body and heading alignment) leads to more rapid perception and as a consequence subjects turn their gaze to the independent motion within the first trial. Humans indeed turned their gaze toward the independently moving figures earlier. Although dogs eventually increased their gaze at the independent motion as well, they initially preferred to look at the chasing pattern^9^. This species difference, however, might not reflect differences in animacy perception, but rather point to a general difference in the visual strategy of dogs and humans indicated by the different frequency in their gaze alternation (humans shifting their look between patterns more often than dogs). Park et al.^21^ also found that dogs display longer fixation and slower saccades than humans, when they presented them with images of human and dog faces and non-face objects. Considering the differences within dog breeds regarding the specific tasks they were selected for, we may gain more information about how selection for specific work/behavioural traits influences animacy perception or their visual strategies.

Thus, we aimed to find out whether selection for different aspects of hunting behaviour affects the preference of chasing motion and the scanning of the stimuli i.e. the visual strategy used. We applied the same method as in our previous studies^8,9^, using isosceles triangles as moving figures. To reduce the influence of experience, we tested companion dogs that were not trained to hunt or to do any sport that involves chasing behaviour. We hypothesised that dogs selected to track and chase game have a stronger inclination to monitor their environment thus they display an overall higher preference to chasing motion, and relatively high frequency of gaze alternation between the two motion patterns. Dogs selected to mark and retrieve downed game (that is, during hunting they initially monitor the environment but later focus on a specific aspect of it), following an initial higher frequency of gaze alternation between the two patterns, should focus their visual attention on the motion that is more interesting to them. We expected that these breeds eventually turn their gaze to the independently moving figures because they are less likely to engage in chasing behaviour (or observe chasing interaction) during hunting. Hypotheses and predictions were made prior to testing.

## Methods

### Ethics

Ethical approval was obtained from the National Animal Experimentation Ethics Committee (PE/EA/1550-5/2019). All methods were carried out in accordance with relevant guidelines and regulations, the experiment was performed in accordance with the EU Directive 2010/63/EU. Owners provided a written informed consent to voluntarily permit their dogs to participate in the study.

### Subjects

The FCI (Fédération Cynologique Internationale) established 10 groups of dog breeds discriminated based on the specific tasks they were selected for. However, from the viewpoint of visual perception and differences in visual strategies we applied a different grouping system.

We tested overall 107 purebred dogs kept as companion animals that were divided into two groups based on the typical type of work the breed was selected for according to breed standards. (1) The Chaser group has breeds that were selected to track and chase game (FCI groups: sighthounds, scent hounds, terriers and pinscher and schnauzers), and in the (2) Retriever group we included dog breeds that were selected to follow, mark and remember downed game and then retrieve it to the hunter (works close to the hunter) (FCI groups: retrievers, flushing dogs and water dogs).

We had to exclude 65 dogs. 57 dogs because they looked at the screen in less than 20% of the time in either trials (Chaser: 23; Retriever: 34). Five dogs because the camera did not capture the dog’s face all the time; three dogs due to technical issues. Compared to the previous studies^8,9^, we changed the exclusion criterion from 10 to 20% regarding the overall looking duration per trial. We decided on this change a priori, because we believe that increasing the minimum looking time improves the method in general (note however, that same criterion would only lead to the exclusion of three dogs in^8^, and none in^9^). In the final analyses, we had 42 dogs (for details, see Table 1 and Supplementary Data S1).

**Table 1:** Details of subjects involved in the final analyses.

Group (N)	Mean age (year) ± SD	Male/female	Breed (N)
Chaser (22)	5.4 ± 4.1	11/10	Beagle (8)
			Jack Russell terrier (4)
			Parson Russell terrier (3)
			Basset hound (2)
			Hanover hound (2)
			German boxer (1)
			Catahoula leopard dog (1)
			Shiba inu (1)
Retriever (20)	3.7 ± 3.0	9/11	Golden retriever (8)
			Labrador retriever (5)
			English cocker spaniel (7)

For more details, see Supplementary Data S1.

### Apparatus

**Figure 1:**
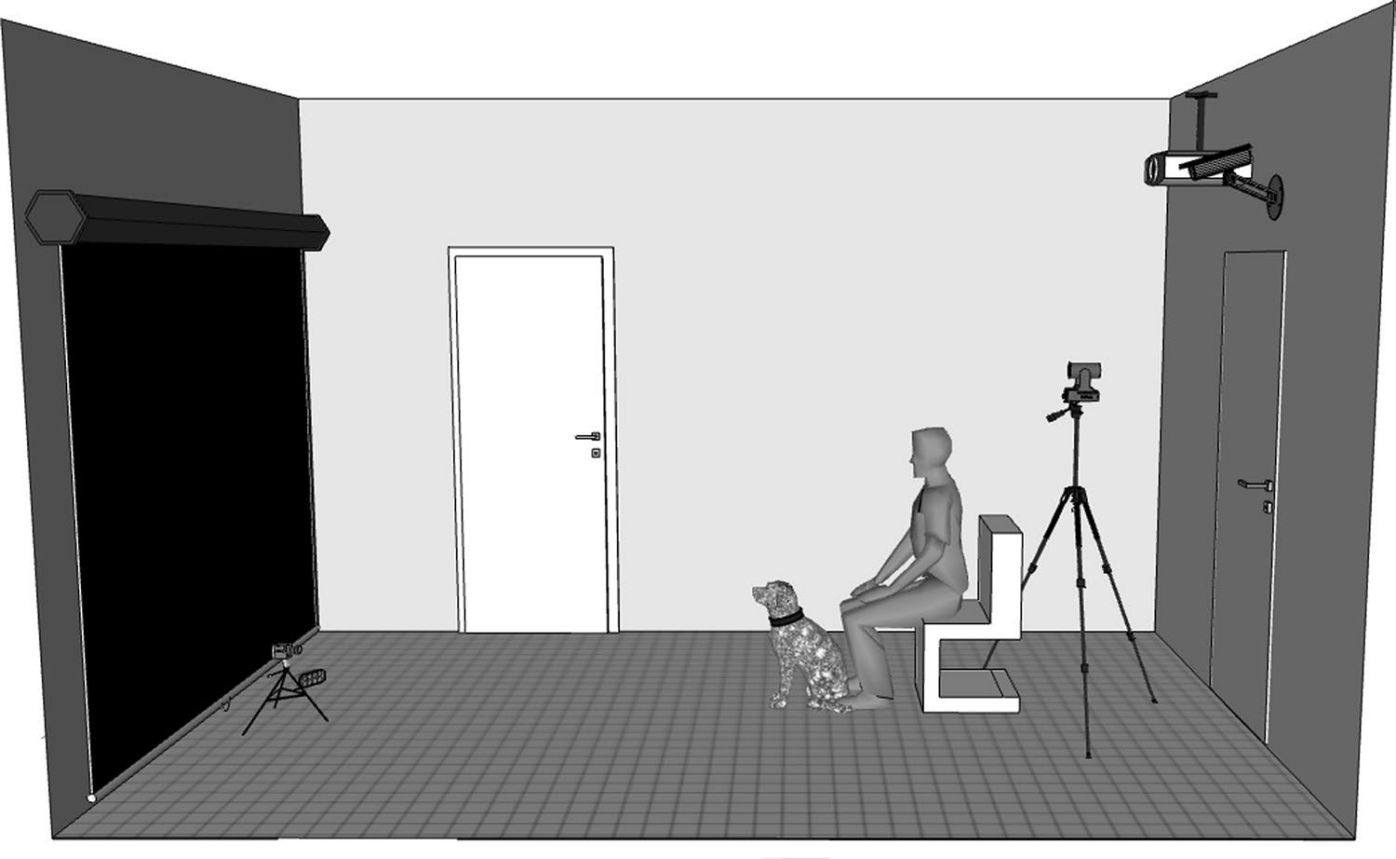
Experimental setup. Subjects sat 2.8 m away from the screen on which the chasing and independent motions were displayed.

Dogs were tested in the 3 m × 5.3 m test room of the Department of Ethology, Eötvös Loránd University, Hungary. The video was displayed on a 2 m × 2.1 m white screen in front of the dog, and the projector was mounted on the ceiling behind the dog. Dogs were sitting on the ground 2.8 m away from the screen (Fig. 1). Infrared LEDs were placed next to the camera directed toward the dogs to improve eye visibility. Audio was displayed by two speakers centred behind the screen to avoid possible asymmetric cues.

Dogs’ face was captured with a 25 frame per second zero lux camera (Sony FDR-AX53) mounted on a compact tripod placed before the screen, equidistant from its sides. The screen was captured by two cameras behind the dog. One of these was attached to the ceiling and was synchronized with the camera in the front automatically in a program. The other camera was mounted on a tripod right behind the owner (Fig. 1). This second camera was used to avoid occasional loss of subjects due to issues with the synchronous camera system.

### Procedure

The owner and the dog entered the room along the experimenter (E), on the door across the screen. The dog could explore the room while E gave instructions to the owner. The owner sat on the chair and held the dog in front of them by the collar or leash, facing the screen. Owners were instructed to look down during the test so they could not see the display on the screen. E adjusted the zero lux camera to capture the dog’s head, and then turned off the light and left the room. E went to the adjacent room and started the video from there. After the video ended, E entered the test room again and the test was finished.

We used the same set of videos as in Abdai et al.^9^, consisting of the following: (1) 2.32 s long audiovisual attention grabber to call the dogs’ attention to the centre of the screen, (2) 10 s stimulus (Trial 1), (3) plain black screen for 3 s, (4) 2.32 s audiovisual attention grabber, and (5) 10 s stimulus (Trial 2) (see Supplementary Video S1). Videos were generated by the ChasingDots program (developed by Bence Ferdinandy^8^). Stimuli were dependent (henceforth ‘chasing’) and independent movement patterns of two white isosceles triangles presented side-by-side, over a plain black background separated by a white vertical line in the middle of the screen. In the independent patterns, one figure was a chaser and the other a chasee from two different chasing patterns; thus the motion dynamics of the chasing and independent patterns were the same. The sides of the chasing and independent patterns were counterbalanced between trials and subjects.

Overall, we had 30 videos and we initially displayed a different video to each subject. Videos displayed to excluded dogs have been used again later for the new subjects. Considering the large number of dogs tested (without exclusion) some videos were displayed to more than one subject (coding of behaviour was continuous but slower than testing, thus it happened that all 30 videos were already used when new subjects were tested). Videos were displayed to the maximum of two dogs within group.

### Behaviour and statistical analyses

Dogs’ behaviour was analysed in Solomon Coder 19.08.02. (^©^András Péter: https://solomon.andraspeter.com/). Behaviour was coded frame-by-frame (25 frames per second) and for every frame it was determined whether the dog looked at the chasing, at the independent motion or away from the screen. Statistical analyses were carried out with R software version 4.1.1^22^ in RStudio version 1.4.1717^23^, and with Python 3.7.6 in Jupyter Notebook 6.0.3.

Inter-coder reliability was assessed on a subsamples of the recordings. A second coder blind to the hypotheses coded 25% of the subjects (dogs used for this analysis are indicated in Supplementary Data S1). For this analysis, we exported the full coding sheets of both coders and checked the correspondence between coders for all data points (i.e. about 500 data points). Inter-coder reliability was tested calculating Cohen’s kappas; analysis indicated acceptable reliability (mean ± SD Cohen’s kappa 0.789 ± 0.120).

Looking duration of subjects was analysed using linear mixed model (LMM; ‘lme4’ package). Residuals of the model were normally distributed after Tukey's ladder of powers transformation (‘rcompanion’ package; lambda 0.6) of the looking duration data (Kolmogorov–Smirnov test: D = 0.039, *p* = 0.956). We estimated the fixed effects of motion pattern (chasing vs independent), trial (Trial 1 vs 2) and group (chaser vs retriever) (three-way interaction). We also tested whether the pattern they looked at first in the specific trial or whether the side on which the chasing pattern was displayed at, had an effect on their looking behaviour. Subjects’ ID was included as a random intercept to control for within-subject comparison. Trial and Pattern were included as random slopes to control for the non-independence of the data. Backward model selection was carried out using drop1 function; selection was based on likelihood ratio test (LRT). LRT of non-significant variables are reported before their exclusion from the model. For significant explanatory variables in the final models, we carried out pairwise comparisons (‘emmeans’ package; Tukey correction) and we report contrast estimates (*β* ± SD).

We also tested the within-trial dynamics of dogs’ gaze at the screen by creating looking-time curves for both patterns within each trial, separately for the two groups. A single point of a curve represents the proportions of time spent looking at the chasing and independent patterns by subjects in the specific group, for every three consecutive frames. Considering that at the beginning of the trials subjects did not look at the stimuli, we only included data points after the proportion values reached 80% of the average proportion of looking time at the stimuli during the specific trial. Linear regression was applied to the data to capture overall trends and estimate slopes (*β* ± SE) (see Supplementary Data S2 for the notebook; data used for this analysis is attached as Supplementary Data S3/Chasers/and Supplementary Data S4/Retrievers/).

We also measured the frequency of shifting the gaze between patterns (irrespective of delays in between) and analysis was carried out using LMM. Residuals of the model was normally distributed after the Tukey's ladder of powers transformation (lambda 0.63) of the frequency of gaze alternation data (Kolmogorov–Smirnov test: D = 0.088, *p* = 0.530). We estimated the fixed effects of trial (Trial 1 vs 2) and group (chaser vs retriever) (two-way interaction), and included the ID of subjects as a random effect. Model selection and reporting of results is the same as in case of the analysis of the looking duration.

## Results

### Looking duration

**Figure 2:**
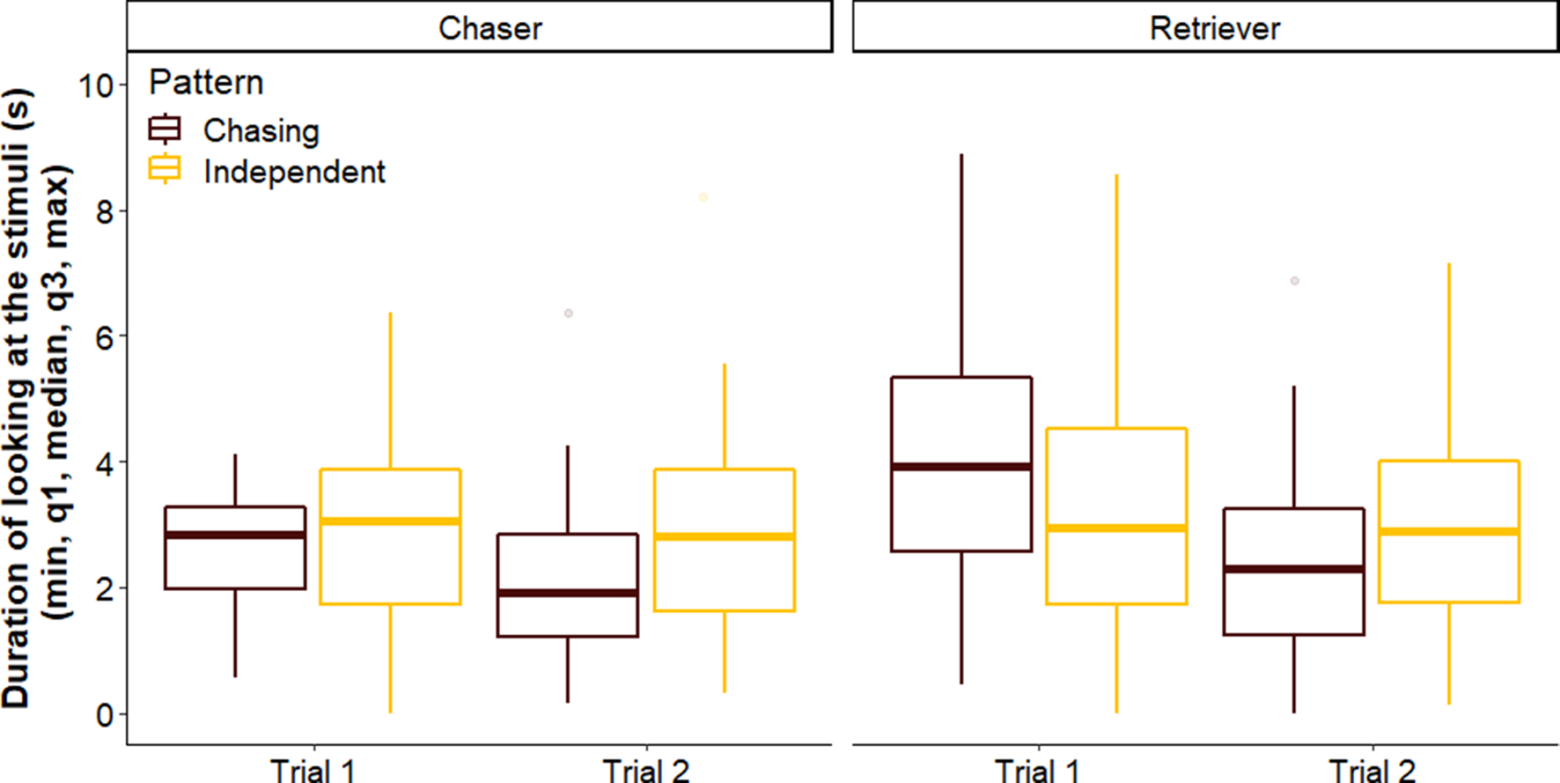
Duration of looking at the chasing and independent patterns in Trials 1 and 2 by dogs in the Chaser and Retriever groups. The boxplots indicate the minimum, the 25th percentile, median, the 75th percentiles and the maximum.

Overall we did not find a difference between breed groups regarding their looking duration toward the patterns across trials (LMM, LRT: Group × Trial × Pattern, $\chi_{1}^{2}$ = 0.392, *p* = 0.531) (Fig. 2). Further, none of the two-way interactions had a significant effect on the looking duration of dogs (Group × Pattern, $\chi_{1}^{2}$ = 0.300, *p* = 0.584; Pattern × Trial, $\chi_{1}^{2}$ = 2.565, *p* = 0.109; Group × Trial, $\chi_{1}^{2}$ = 2.028, *p* = 0.155). We also did not find an overall difference between Chaser and Retriever dogs, or in their looking duration toward the patterns (Group, $\chi_{1}^{2}$ = 2.926, *p* = 0.087; Pattern, $\chi_{1}^{2}$ = 0.503, *p* = 0.478). However, dogs in general looked longer at the stimuli in Trial 1 than in Trial 2 (LRT: Trial, $\chi_{1}^{2}$ = 5.011, *p* = 0.025; Trial 1 vs Trial 2: β ± SE = 0.235 ± 0.107, *p* = 0.033).

The side on which the chasing pattern was displayed at, or the pattern dogs looked at first had no effect on their looking duration either (LRT: Side, $\chi_{1}^{2}$ = 0.033, *p* = 0.855; First look, $\chi_{1}^{2}$ = 1.710, *p* = 0.191).

### Within trial dynamics of looking at the stimuli

**Figure 3:**
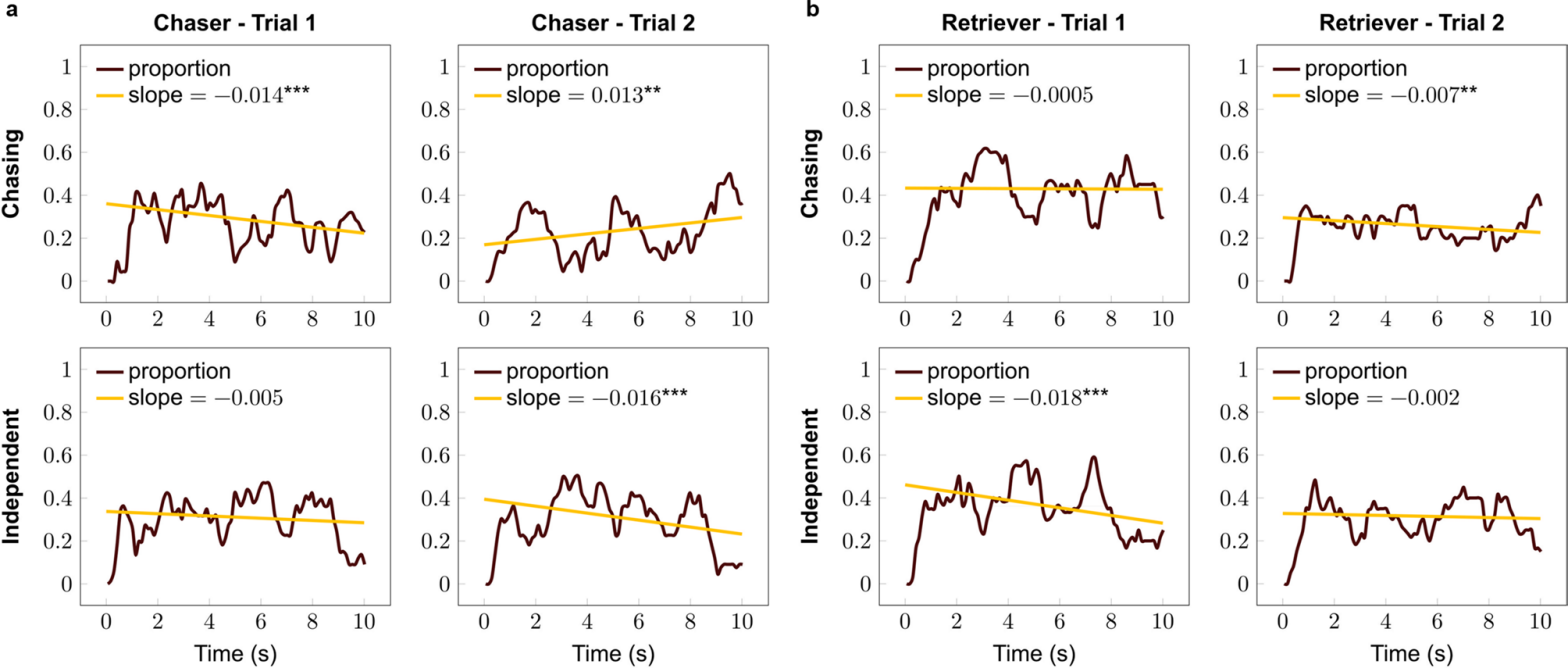
Proportions of looking at the chasing and independent patterns in the (**a**) chaser and (**b**) retriever groups. *p < 0.05, ***p < 0.001.

The looking duration of subjects did not show any difference between breed groups; however, this analysis does not provide information about the change in the looking time toward the specific stimuli. Thus we also carried out a more descriptive analysis about the dynamics of the looking behaviour at each pattern within trials. We found that Chaser breeds reduced their look toward the chasing motion in Trial 1 while keeping their visual attention on the independent motion; however, in Trial 2 they increased their look toward the chasing, and decreased it toward the independent motion (Fig. 3a). Retrievers decreased their look toward the independent motion in Trial 1 while keeping their look constant on the chasing pattern, which switched for the second trial, i.e. their look decreased toward chasing while keeping constant at the independent motion (Fig. 3b). For detailed results, see Table 2.

**Table 2:** Within-trial dynamics of looking at each pattern within Trial 1 and 2 (linear regression).

Group	Trial	Pattern	Direction	*β* ± SE	*p* value
Chaser	Trial 1	Chasing	↓	− 0.014 ± 0.004	< 0.001
		Independent	NC	− 0.005 ± 0.004	= 0.219
	Trial 2	Chasing	↑	0.013 ± 0.005	= 0.006
		Independent	↓	− 0.016 ± 0.004	< 0.001
Retriever	Trial 1	Chasing	NC	− 0.0005 ± 0.004	= 0.900
		Independent	↓	− 0.018 ± 0.004	< 0.001
	Trial 2	Chasing	↓	− 0.007 ± 0.002	= 0.003
		Independent	NC	− 0.002 ± 0.003	= 0.439

In case of direction, ↓ indicates reducing and ↑ increasing visual attention, *NC* indicates no change.

### Gaze alternation between patterns

**Figure 4:**
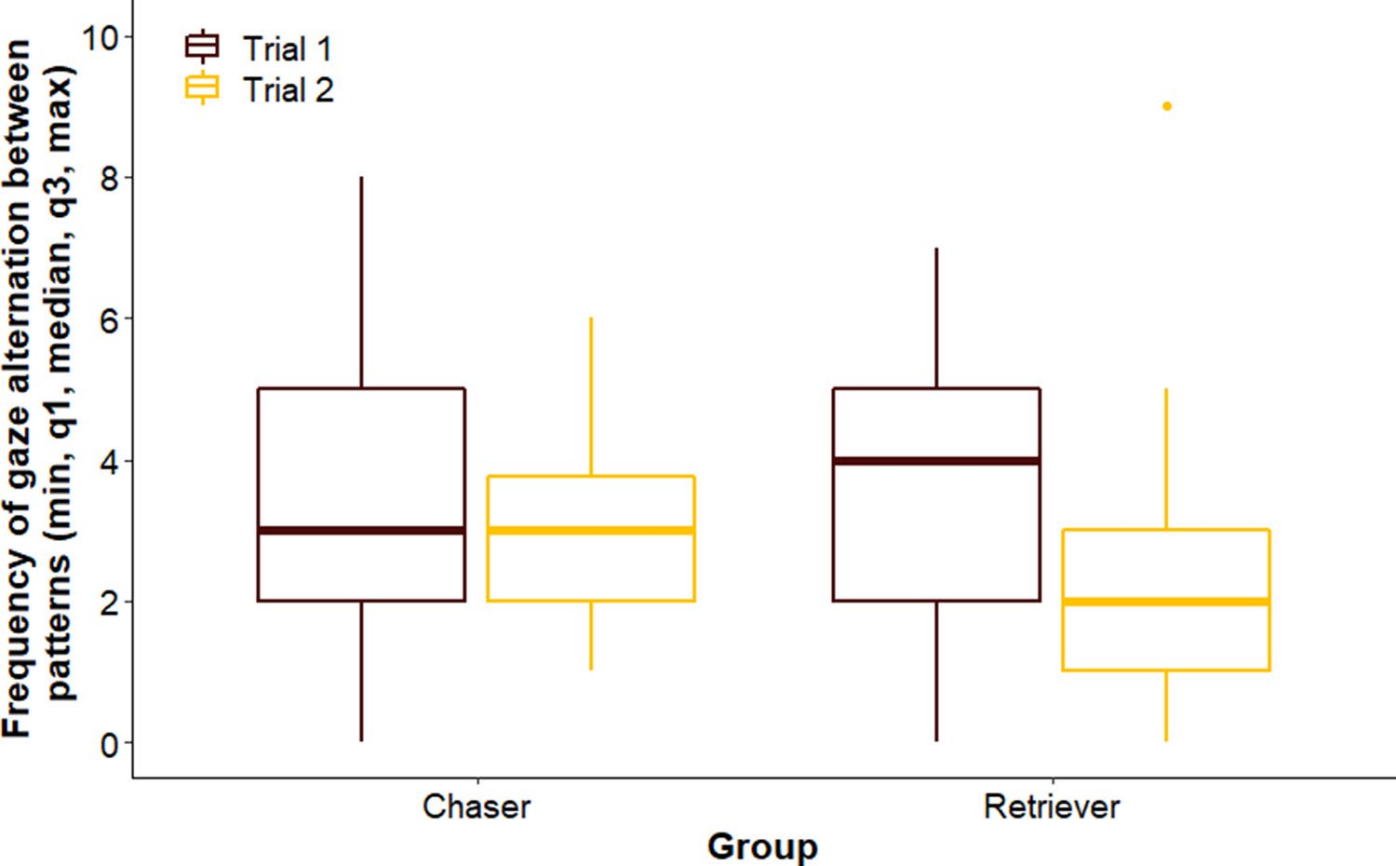
Frequency of gaze alternation of dogs in the different groups.

We did not find difference between groups across trials (LMM, LRT: Group × Trial, $\chi_{1}^{2}$ = 0.307, *p* = 0.580); or among breed groups overall (Group, $\chi_{1}^{2}$ = 0.088, *p* = 0.767), but dogs shifted their gaze between patterns more often in Trial 1 than in Trial 2 (Trial, $\chi_{1}^{2}$ = 6.384, *p* = 0.012; Trial 1 vs Trial 2: β ± SE = 0.450 ± 0.174, *p* = 0.013) (Fig. 4).

## Discussion

We did not find overall differences between dogs depending on the type of work they were originally selected for. At the descriptive level, we can observe that dogs selected to track and chase prey seemed to quickly perceive the chasing motion, therefore they turned their gaze toward the independent motion already in the first trial which is similar to our findings in adult humans, using the same stimuli^9^. However, instead of continue to look at the independently moving figures as expected based on our previous studies^8,9^, Chaser dogs regained their interest in the chasing pattern. Regarding the dynamics of their look, Retrievers initially decreased their look toward the independent, whereas later toward the chasing pattern. These patterns, however, are difficult to interpret, since we do not have statistical evidence that the observed differences in the slopes are not random variations of an identical pattern.

Selection for specific behaviour also seemed to have no influence on the gaze alternation of dogs between patterns. Considering that the visual field of dogs is larger than that of humans^17,19^, it might explain why they do not need fast saccades and short fixation in order to scan the environment efficiently^9,21^. The lack of difference between breed groups suggests that this trait in dogs drive their looking behaviour rather than the more specific differences between breeds.

It has been proposed that animacy perception is an evolutionarily ancient mechanism^5^; however, a comprehensive comparative research is missing to understand what are the underlying genetic or environmental factors influencing the emergence of animacy perception, and whether different species rely on similar motion cues. Previous results in newly hatched chicks and new born human infants indicate that the perception is present from birth in at least two, evolutionary distant species that seem to be sensitive to similar cues (self-propelledness)^3–6^. Direct comparison of adult dogs and adult humans also indicate that both species discriminate between chasing and independent motion patterns displayed by inanimate objects, although differences were also revealed^8,9^. Although in the present study, within-trial dynamics revealed different looking patterns in the two groups, we did not find a significant difference between the looking durations of Chaser and Retriever dogs. Thus, overall it seems that selection for specific tasks within hunting does not influence the basic mechanisms of animacy perception in dogs.

Considering that both dogs’ looking duration toward the screen and the frequency of gaze alternation decreased for Trial 2, we suggest that dogs might lost their interest in the displayed video quickly in the present experiment. Although video display of stimuli is used widely in dog research (e.g.^24–26^), in general dogs are not used to watching screens (e.g. TV) (see also^8^). We had to exclude a high percentage of our subjects because they looked at the screen for less than 2 s (Chaser: 48%, Retriever: 57%) (cf.^8,9^ in which 1 s was applied as criterion). But without further experiments we cannot confirm whether this was caused by lack of interest in display in general or was tied to the displayed stimuli itself. Although in previous studies^8,9^ dogs had to be excluded for similar reasons, we lost less subjects (note that raising the exclusion criterion of the minimum looking duration per trial would have led to the exclusion of three^8^ or no dogs^9^ in these studies, thus this change cannot be responsible for the difference). We do not know whether this is by chance or there is an underlying reason, for example, due to the changed lifestyle of owners and thus their dogs during the pandemic, or the tested breeds are less likely to look at screens.

We would like to address some limitations of the present study. The relatively low number of subjects and lack of diversity of breeds within groups call for caution when drawing strong conclusions. (1) As the high percentage of excluded dogs indicate, subject number can only be increased by testing about twice as many dogs as needed. (2) Considering that some dog breeds are more popular than others in Hungary (although it is probably true in other countries as well), this limits our options to test multiple breeds within groups and multiple subjects within specific breeds. (3) To be able to detect such differences among breed groups, sample size might be increased in this specific scenario.

In summary, our results show that selection for specific tasks within hunting dogs does not influence their chasing perception or their visual strategy in this scenario. However, dogs seemed to rapidly lose interest in the displayed video. Thus, we suggest that novel methodological approach may be used in the future to study animacy perception by applying artificial agents that demonstrate the motion cues eliciting this perception in real life instead of video recordings (see^20^).

## Supplementary Information

Supplementary Information 1.

Supplementary Information 2.

Supplementary Information 3.

Supplementary Information 4.

Supplementary Video S1.

## Data Availability

All data are available as supplementary information.
